# Remodeling of the pulmonary artery in idiopathic pleuroparenchymal fibroelastosis

**DOI:** 10.1038/s41598-019-57248-3

**Published:** 2020-01-15

**Authors:** Yoshiaki Kinoshita, Hiroshi Ishii, Hisako Kushima, Takeshi Johkoh, Hidetake Yabuuchi, Masaki Fujita, Kazuki Nabeshima, Kentaro Watanabe

**Affiliations:** 1 Department of Respiratory Medicine, Fukuoka University Hospital, Fukuoka, Japan; 2 Department of Pathology, Fukuoka University School of Medicine and Hospital, Fukuoka, Japan; 3 Department of Respiratory Medicine, Fukuoka University Chikushi Hospital, Fukuoka, Japan; 4 Department of Radiology, Kansai Rosai Hospital, Hyogo, Japan; 5 Department of Health Sciences, Faculty of Medical Sciences, Kyushu University, Fukuoka, Japan; 6 Department of Respiratory Medicine, Nishi Fukuoka Hospital, Fukuoka, Japan

**Keywords:** Respiratory distress syndrome, Chronic inflammation

## Abstract

Idiopathic pleuroparenchymal fibroelastosis (IPPFE) is a rare subtype of idiopathic interstitial pneumonia that consists of alveolar septal elastosis and intra-alveolar collagenosis, which is predominantly located in the upper lobes. The aim of this study was to examine the remodeling of the pulmonary arteries in patients with IPPFE. This study included 18 patients with IPPFE, 24 patients with idiopathic pulmonary fibrosis (IPF), and 5 patients without pulmonary disease as controls. We selected muscular pulmonary arteries and calculated the percentage of the thickness of each layer of the wall (intima, media, and adventitia) in relation to the external diameter. We also quantified the percentage of areas of elastic fiber in the media divided by the whole area of the media (medial elastic fiber score). The percentage of adventitial thickness in IPPFE was significantly higher than that in IPF and in control lungs. The percentage of medial thickness did not differ statistically between IPPFE and IPF. However, the medial elastic fiber score in IPPFE was also significantly larger than that in IPF and control lungs. These results suggest that collagenous thickening of the adventitia and medial elastosis are distinct histological features in the muscular pulmonary arteries of patients with IPPFE.

## Introduction

Idiopathic pleuroparenchymal fibroelastosis (IPPFE) is a rare subtype of idiopathic interstitial pneumonias that consists of elastofibrosis involving the lung parenchyma and pleura, predominantly located in the upper lobes^1–3^. Histologically, pleuroparenchymal fibroelastosis (PPFE) is characterized by septal elastosis and intra-alveolar collagenosis with or without collagenous fibrosis of the visceral pleura^1,4–6^.

Patients with chronic lung fibrosis, such as idiopathic pulmonary fibrosis (IPF), are prone to pulmonary hypertension (PH), and the complication of PH has a negative impact on the prognosis of affected patients^7,8^. In IPF, remodeling of the pulmonary arteries is characterized by luminal stenosis, which results from the thickening of the intima and the media^9–11^. Meanwhile, no histological studies have fully elucidated the characteristics of the remodeling of pulmonary arteries in IPPFE.

From our past experience in diagnosing IPPFE patients, we noticed that the pulmonary arteries of IPPFE showed some distinct patterns of remodeling: medial thickening with elastosis and adventitial collagenous thickening. The aim of this study was to investigate the histological patterns of remodeling of the pulmonary arteries of IPPFE patients.

## Results

### Patient characteristics

The patients’ characteristics are summarized in Table 1. In IPPFE, pack-years smoking, body mass index, and serum KL-6 levels were significantly lower than in IPF, and there was a significant difference in the smoking status of IPPFE and IPF. The forced vital capacity, both absolute values and % predicted values, in IPPFE were significantly lower than those in IPF. % predicted values of the residual volume and (residual volume)/(total lung capacity) in IPPFE were significantly higher than those in IPF.

**Table 1:** Clinical characteristics.

Factor	IPF (n = 24)	IPPFE (n = 18)	P value^†^
Age, years	65.2 ± 10.2	59.3 ± 13.2	0.111
Gender, male/female, n	17/7	9/9	0.21
Smoking status, current/former/never, n	4/15/5	1/4/13	0.003
Pack-years smoking	33.5 ± 42.7	7.94 ± 14.9	0.02
Body mass index, kg/m^2^	22.4 ± 2.98	16.4 ± 3.00	<0.001
Serum KL-6 levels, U/mL*	1127 ± 777	497 ± 214	0.003
Respiratory function parameters*			
FVC, ml	2364 ± 880	1751 ± 810	0.026
FVC, % predicted	72.6 ± 19.0	54.8 ± 23.4	0.01
RV, % predicted	56.9 ± 21.4	91.9 ± 37.0	0.001
RV/TLC, % predicted	86.5 ± 26.2	138 ± 45.6	<0.001
DL_CO_, % predicted	61.0 ± 26.1	70.2 ± 25.7	0.31
DL_CO_/VA, % predicted	83.9 ± 29.5	87.1 ± 31.9	0.767

*The respiratory functional parameters and serum KL-6 levels were measured less than one year prior to tissue collection.

^†^p values were calculated for the comparison of IPF and IPPFE patients.

IPF, idiopathic pulmonary fibrosis; IPPFE, idiopathic pleuroparenchymal fibroelastosis; KL-6, Kerbs von Lungren-6 antigen; FVC, forced vital capacity; RV, residual volume; TLC, total lung capacity; DL_CO_, diffusing capacity of the lung for carbon monoxide; VA, alveolar volume.

### The diameter and thickness of the pulmonary artery wall

The diameter and thickness of the pulmonary artery wall are summarized in Table 2. The external diameter was equivalent among the control, IPPFE and IPF lungs.

**Table 2:** The diameter and thickness of the muscular pulmonary artery wall.

Factors	Normal (n = 5)	IPF (n = 24)	IPPFE (n = 18)	P value*
External diameter, μm	389 ± 26.6	342 ± 53.8	351 ± 71.0	0.632
Luminal diameter, %	59.0 ±± 2.01	41.0 ± 11.9	37.3 ± 7.80	0.261
Intimal thickness, %	6.84 ± 2.07	19.4 ± 8.95	16.9 ± 6.65	0.326
Medial thickness, %	15.0 ± 1.42	19.8 ± 4.46	19.5 ± 3.25	0.859
Adventitial thickness, %	19.0 ± 1.15	19.6 ± 4.07	26.0 ± 7.27	0.001

IPF, idiopathic pulmonary fibrosis; IPPFE, idiopathic pleuroparenchymal fibroelastosis.

*p values were calculated for the comparison of IPF and IPPFE patients. (Student’s t-test).

The percentage of medial thickness in both IPF (19.8 ± 4.46) and IPPFE (19.5 ± 3.25) was significantly higher in comparison to the control lungs (15.0 ± 1.42) (*p* = 0.028 and 0.002, respectively). The percentage of medial thickness did not differ statistically between IPPFE and IPF (*p* = 0.859).

The percentage of adventitial thickness in IPPFE (26.0 ± 7.27) was significantly higher than that in IPF (19.6 ± 4.07, *p* = 0.001) and in control lungs (19.0 ± 1.15, *p* = 0.003). Meanwhile, the percentage of adventitial thickness did not differ statistically between the control lungs and IPF (19.0 ± 1.15 vs. 19.6 ± 4.07, *p* = 0.718).

The diameter and thickness of the wall in the pulmonary arteries, categorized by vessel size, are summarized in Supplementary Dataset. The percentage of intimal thickness did not differ statistically between IPPFE and IPF. The percentage of adventitial thickness in IPPFE was significantly higher than that in IPF when the groups categorized by vessel size were compared. The percentage of medial thickness in IPF became significantly larger than that in IPPFE (20.7 ± 5.05 vs. 17.0 ± 4.36, *p* = 0.023) when arteries of 501–1000 μm in external diameter were compared.

### The medial elastic fiber score

**Figure 1:**
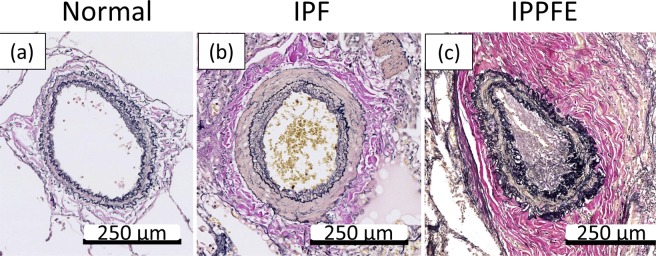
Representative examples of Elastica van Gieson-stained sections of the pulmonary arteries from the control lung (**a**), and patients with IPF (**b**), and IPPFE (**c**). (**b**) shows medial hypertrophy with muscularization, and (**c**) shows adventitial collagenous thickening and medial elastosis including thickening of the inner and outer elastic lamina.

**Figure 2:**
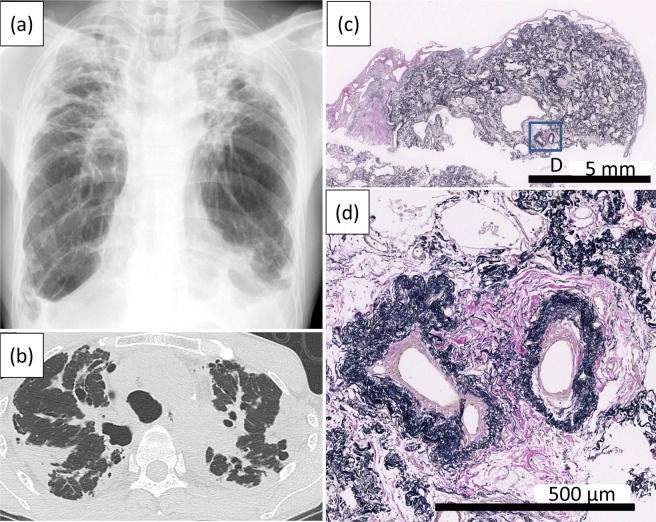
A chest radiograph (**a**) and computed tomography scan (**b**) of an IPPFE patient showing subpleural wedge-shaped consolidations with upper lobe-predominance. An Elastica van Gieson-stained section (**c**) shows the subpleural accumulation of elastic fibers. The pulmonary arteries show the thickening of the adventitia and media, which are totally replaced by elastic fibers (**d**).

The medial elastic fiber score in IPF (38.6 ± 11.0) was significantly higher than that in the control lungs (27.9 ± 5.31) (*p* = 0.033). The medial elastic fiber score in IPPFE (46.8 ± 13.5) was also significantly higher than that in IPF (38.6 ± 11.0, *p* = 0.036) and that in control lungs (27.9 ± 5.31, *p* = 0.005). Representative examples of Elastica van Gieson (EVG)-stained sections of pulmonary arteries are shown in Figs. 1 and 2.

### Clinical PH and histologic vasculopathy

Among the 18 patients with IPPFE, right heart catheterization was performed in one patient and echocardiography in 16 patients. Among the 24 patients with IPF, right heart catheterization was performed in seven patients and echocardiography in 22 patients. Mean pulmonary arterial pressure (mmHg) in the IPPFE patients (n = 16) was equivalent to that in the IPF patients (n = 22) (23.7 ± 11.3 vs. 22.7 ± 9.49, *p* = 0.772). Among the 16 IPPFE patients who were assessed by echocardiography and/or right heart catheterization, four patients (25%) were complicated with clinical PH; one patient was diagnosed by right heart catheterization and three were diagnosed by transthoracic echocardiography. Among the 22 IPF patients who were assessed by echocardiography and/or right heart catheterization, four patients (18.2%) were complicated with PH that was diagnosed by right heart catheterization. No patients had underlying conditions that could lead to PH including left heart disease, sleep-disordered breathing, chronic high-altitude exposure, chronic thromboembolic pulmonary hypertension, blood disorders, sarcoidosis, and histiocytosis^12^.

When we divided the patients with IPPFE into two groups with or without PH, medial elastic fiber scores in IPPFE patients with PH (n = 4) were not significantly different from those without PH (n = 14) [median (interquartile range): 45.2% (40.0–55.2) vs. 36.9% (17.8–45.2), *p* = 0.327]. Similarly, the percentage of adventitial thickness in IPPFE patients with PH (n = 4) were not significantly different from that without PH (n = 14) [27.1% (25.4–32.3) vs. 24.1% (21.2–26.7), *p* = 0.277].

The Heath–Edwards score in IPPFE was equivalent to that in IPF (3.00 ± 0.59 vs. 2.96 ± 0.55, *p* = 0.816).

### The association between pulmonary artery remodeling and the prognosis of IPPFE

We divided 12 patients with IPPFE who had been diagnosed by surgical lung biopsy into two groups by the median of medial elastic fiber score, and also divided them into two groups by the median of the percentage of the adventitial thickness (high group vs. low group), and compared the survival curve between the groups. However, survival was not significantly different between groups either when divided by the medial elastic fiber score or when divided by the percentage of the adventitial thickness (*p* = 0.147 and 0.468, respectively).

## Discussion

In the present study, we showed that medial elastosis and collagenous thickening of the adventitia were distinct features of IPPFE. Although septal elastosis and intra-alveolar collagenosis are known as unique and distinct histologic features of PPFE^1,4–6^, we showed that elastosis and collagenosis were present in the pulmonary arteries as well as the alveolar walls and alveolar spaces in IPPFE.

The muscular arteries usually measure between 100 and 1000 μm in external diameter and their media is composed of smooth muscle cells surrounded by elastic laminas^13–15^. Among patients with pulmonary arterial hypertension, remodeling of the pulmonary arteries in the muscular arteries includes muscular hypertrophy of the media, intimal thickening by cellular proliferation and/or fibrosis, plexiform lesions, and fibrinoid necrosis^14–16^. Usual interstitial pneumonia (UIP) patterns—especially in IPF associated with PH—were previously reported to be associated with the thickening of the smooth muscle cell layer and proliferative intimal lesions^10,11^. However, elastosis in the media has not been described as a morphological change in the muscular arteries of patients with PH or pulmonary fibrosis.

Elastic fibers originally provide recoil tension to restore the structure of vascular tissues or organs and are essential for their function^17^. However, once elastic fibers are produced in a disorderly manner, they diminish the proper function of organs, leading to—for instance—restrictive ventilatory dysfunction in PPFE patients^6,18^. Regulators of elastogenesis include transforming growth factor-β, fibroblast growth factor-2, epidermal growth factor, and insulin-like growth factor-1^17^. It is unknown whether these mediators contribute to the formation of subpleural fibroelastosis or medial elastosis in PPFE. Further investigation will be needed to elucidate the pathogenesis of medial elastosis in PPFE.

In this study, we found another unique histological feature in the muscular pulmonary arteries of PPFE: collagenous thickening of the adventitia. Besides septal elastosis, intraalveolar collagenosis is another essential histological feature of PPFE^1,4–6^. Previously, we quantitatively compared collagen fibers between IPF, PPFE, and control lungs and showed that the amount of collagen fibers in IPF and PPFE was increased to an equivalent degree in comparison to control lungs^19^. However, adventitial collagenous thickening of the muscular arteries is not common in IPF^9–11^. Collagenous thickening of the adventitia in the pulmonary arteries may also be a distinct histological feature in IPPFE.

Patients with chronic lung disease are originally prone to PH^7,8^. PH is a common complication of IPF, and the prevalence varies according to the severity of the disease^20–22^. PH has been found in 19% of IPF patients with a mildly to moderately restricted respiratory function^20^ and in 38–46% of those with advanced stage disease^21,22^. Although the prevalence of PH in IPPFE patients has not been investigated, there are a few case reports of PPFE patients with PH^23–25^. In this cohort, we found that four of 16 IPPFE patients (25%) were complicated with clinical PH. Thus, physicians should pay attention to the complication of PH in the clinical course of IPPFE.

In this study, medial elastic fiber scores and the percentage of adventitial thickness in patients with IPPFE did not affect survival or the complication of PH. That might be partly due to a small number of patients enrolled in the present study. Although the combined thickness of the intima and the media appears to be correlated with pulmonary artery pressures or vascular resistance^16^, there was a marked overlap of the fractional thickness of media and adventitia between lungs obtained from patients with pulmonary arterial hypertension and control lungs^26^. In addition, it is little known whether or not changes in major components of media from smooth muscle cells to elastosis influence pulmonary artery pressures or vascular resistance directly.

The present study was associated with several limitations. First, this was a retrospective study performed in a single center, and the number of patients was relatively small. Second, fixation of the lung with distension avoids crenation of the elastic lamina of the pulmonary arteries, which may induce “false” medial hypertrophy^27^. Although we histologically confirmed that all lungs were satisfactorily inflated, it was difficult to standardize the degree of inflation. Third, although right heart catheterization is still the gold standard for the diagnosis of PH^12^, right heart catheterization was only performed in one of the four IPPFE patients with PH in this study. Echocardiography is a widely applied non-invasive diagnostic tool for the assessment of PH^12^. Patients who fulfilled the echocardiographic criteria for PH in this study had a high probability of having PH^12^. However, the clinical diagnosis of PH by echocardiography alone may be insufficient.

In conclusion, the histological pattern of remodeling in the muscular pulmonary arteries differed between IPPFE and IPF; medial elastosis and collagenous thickening of the adventitia were distinct features of IPPFE.

## Materials and Methods

### Subjects

We retrospectively reviewed our clinical records between 1995 and 2017, and selected 18 patients with IPPFE who had been clinically diagnosed and whose diagnosis had been confirmed by a pathological examination in Fukuoka University Hospital^1,28–30^. Twelve patients underwent a surgical lung biopsy and 6 were examined at autopsy.

As a control for IPPFE, we reviewed clinical and histologic records from 2006 to 2017, and selected 30 consecutive patients who had been clinically diagnosed as IPF and histologically diagnosed as UIP in Fukuoka University Hospital. According to the diagnostic criteria for IPF in the 2018 ATS/ERS/JRS/ALAT Clinical Practice Guideline^31^, we re-evaluated and classified the 30 patients radiologically and histologically in the following sections *Radiologic categories of UIP* and *Histologic categories of UIP*.

### Radiologic categories of UIP

The 30 patients underwent chest computed tomography (CT) using either one of the two CT scanners, Aquilion64 (Toshiba Medical Systems) or Aquilion ONE (Toshiba Medical Systems). Scanning parameters included automatic tube current adoption, 120-kV tube voltage, 64 × 0.5 mm collimation, and pitch of 0.8. All images were reconstructed into axial images with a 1–2 and 5 mm slice thickness at 5 mm intervals and were photographed at window settings appropriate for viewing the lung parenchyma (window level, −600 HU; window width, 1500 HU). Chest CT images were independently evaluated by two experienced pulmonary radiologists (TJ and HY) according to High-Resolution Computed Tomography (HRCT) Scanning Patterns in the updated guidelines for IPF^31^. Disagreements were resolved by consensus. The fibroses of the 30 patients were radiologically classified as UIP (n = 8), probable UIP (n = 10), indeterminate for UIP (n = 9) and alternative diagnosis (n = 3).

### Histologic categories of UIP

Samples were fixed in 10% formalin and embedded in paraffin. Tissue sections (thickness: 4 μm) mounted on glass microscope slides were stained with hematoxylin-eosin, EVG, and Masson’s trichrome. The histologic patterns of fibrosis in the 30 IPF patients were decided according to Histopathology Patterns and Features in the updated guidelines for IPF^31^. Patterns were independently evaluated by two experienced pulmonary pathologists (KW and YK), and disagreements were resolved by consensus. The fibroses were histologically classified as UIP (n = 21), probable UIP (n = 7), and indeterminate for UIP (n = 2). Nine cases that were radiologically indeterminate for UIP were histologically classified as UIP in seven patients, probable UIP in one, and indeterminate for UIP in one.

### IPF as a control for IPPFE defined by the 2018 Guideline^31^

Finally, 24 patients were diagnosed as having IPF by the specific combinations of HRCT patterns and histopathology patterns in the updated guideline for IPF^31^, and data of the 24 patients were selected as a control for IPPFE (autopsy, n = 1; pneumonectomy for lung transplantation, n = 6; and surgical lung biopsy, n = 17).

### Normal control for IPPFE

Autopsied samples obtained from apparently normal lungs of five consecutive patients who had died of causes other than pulmonary diseases were used as a normal control.

### Clinical and respiratory function data

Clinical data were abstracted from the patients’ medical records. The respiratory function data were obtained as described in Supplementary Data 1.

### The diameter and thickness of the pulmonary artery walls

For patients whose histologic specimens were obtained from surgical lung biopsy, we measured the diameter and the thickness of the wall (intima, media, and adventitia) in the pulmonary arteries in all specimens. We examined four representative histologic specimens for pneumonectomized or autopsied patients: two specimens each from resected upper and lower lobes, and one specimen each from bilateral upper and lower lobes, respectively.

**Figure 3:**
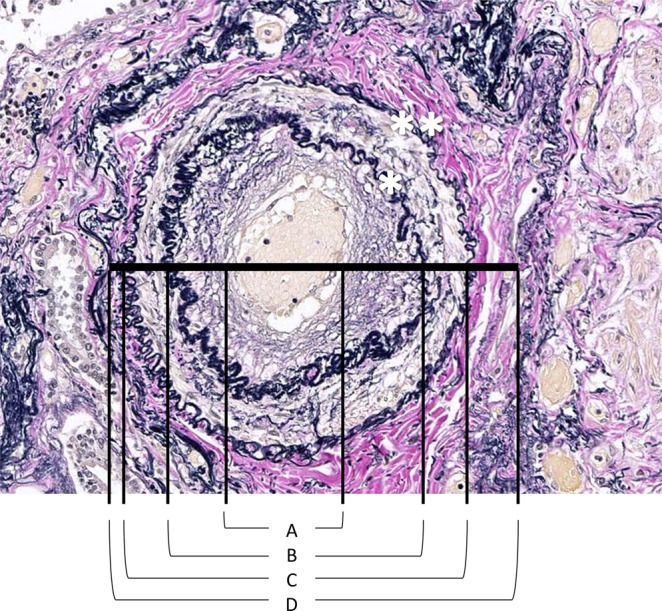
A representative example of an Elastica van Gieson-stained pulmonary artery section showing the luminal diameter (**A**), intimal diameter (**B**), medial diameter (**C**), and external diameter (**D**). *Internal elastic lamina, **external elastic lamina.

EVG-stained slides of the selected specimens were scanned and converted to whole-slide images, also known as a virtual slide, with a NanoZoomer 2.0-RS (Hamamatsu Photonics, Hamamatsu, Japan). We evaluated all pulmonary arteries in the selected specimens that satisfied the following conditions^32^: located adjacent to a bronchus or bronchiole, having two or more elastic laminas, having an external diameter of 150–1000 μm, having a true cross-section in which the length of the longest external diameter was less than twice the shortest external diameter, and having a well-defined adventitia. The external diameter was defined as the length connecting the external edge of the adventitia on one side to that on the other on the shortest line of the cross-section of the pulmonary arteries (Fig. 3).

After measuring the external diameter, the luminal diameter and the thicknesses of the intima, media, and adventitia were then measured in the same pulmonary artery. A representative example is shown in Fig. 3. The luminal, intimal, medial, and external diameters are shown as (A), (B), (C), and (D), respectively. The intimal, medial, and adventitial thickness were calculated by (B) minus (A), (C) minus (B), and (D) minus (C), respectively. We then calculated the percentage of the luminal diameter and the intimal, medial, and adventitial thickness in relation to the external diameter as follows: % luminal diameter = luminal diameter/external diameter × 100; % intimal thickness = intimal thickness/external diameter × 100; % medial thickness = medial thickness/external diameter × 100; and % adventitial thickness = adventitial thickness/external diameter × 100. These parameters were averaged in each patient. The data were also evaluated separately in three groups categorized by the length of the external diameter of the pulmonary artery: 151–250 μm; 251–500 μm; and 501–1000 μm because the fractional thickness of each vessel coat may differ according to the vessel size^9,13,15^.

### Quantification of the elastic fibers in the pulmonary artery media

The histological changes in PH initially occur in the pulmonary arteries of <500 μm in diameter and the media in the pulmonary arteries of <250 μm was quite thinner to be evaluated^14^. Thus, we randomly selected 10 pulmonary arteries with external diameters ranging from 251 to 500 μm in each patient.

**Figure 4:**
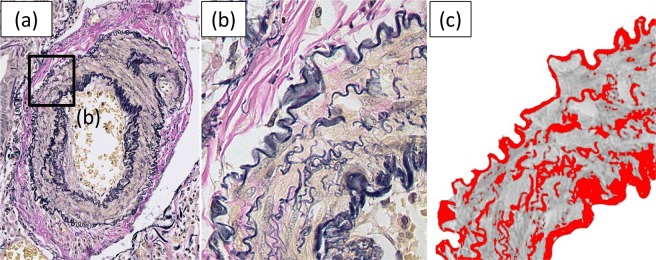
A representative example of an Elastica van Gieson-stained pulmonary artery section (**a** and **b**, inset of **a**). The area of the media in (**b**) was extracted and binarized (**c**). The red area in (**c**) shows elastic fibers. The elastic fiber score, (red area)/(gray area + red area)x100, was calculated to be 31.9%.

The media of the pulmonary arteries was defined as the area between the outer elastic lamina and the inner elastic lamina including the inner edge of the inner elastic lamina and the outer edge of the outer elastic lamina on EVG-stained sections. The area of the media was selected manually using Adobe Photoshop Elements 12 (Adobe Systems, San Jose, CA, USA). The medial elastic fiber score (%) was defined as the number of pixels of the elastic fiber in the media divided by the number of pixels of the media, multiplied by 100 (Fig. 4). On the EVG-stained slides, the medial elastic fiber scores were assessed using the ImageJ 1.49 v software program (National Institutes of Health, Bethesda, MD, USA) with the same threshold, as described previously^19,33^. The scores were then averaged in each patient.

### Clinical PH and histologic vasculopathy

Clinical PH was diagnosed based on the 2015 ESC/ERS guidelines^12^: (1) a mean pulmonary arterial pressure of ≥25 mmHg by right heart catheterization; or (2) a peak tricuspid regurgitation velocity of >3.4 m/s or 2.9–3.4 m/s with the presence of other signs indicative of PH on transthoracic echocardiography. In Doppler echocardiography, the mean pulmonary arterial pressure and the tricuspid regurgitation velocity were estimated as previously described^34,35^. When the patients had undergone both Doppler echocardiography and right heart catheterization, we used the parameters measured by right heart catheterization.

We evaluated histological vasculopathy in the pulmonary arteries using the Heath–Edwards classification^14^.

### Statistical analysis

Continuous data are shown as the group means (standard deviation), and categorical data are shown as the number (percentage) in the group. Fisher’s exact test was used to compare categorical variables. Differences between groups were assessed using Student’s *t*-test for unpaired data or the Mann–Whitney U test for continuous variables. Kaplan-Meier survival curves were plotted, and the differences of the survival period beginning at the time of biopsy were compared between two groups using log-rank test. A P value of < 0.05 was considered to indicate statistical significance. All statistical analyses were performed using the R software program (version 3.2.2; R Foundation for Statistical Computing, Vienna, Austria).

The Fukuoka University Medical Ethics Review Board (FU-MERB) approved the study protocol and waived the requirement for informed consent (approval number: 217M037).

### Sources of support

This study was partially supported by a grant from the Ministry of Health, Labor and Welfare of Japan awarded to the Study Group on Diffuse Pulmonary Disorders, Scientific Research/Research on Intractable Diseases.

## Supplementary information

Supplementary Table 1. The diameter ant thickness of the wall of the pulmonary arteries categorized by vessel size.

Supplementary data 1. Respiratory function data.
