# Supplementary material for: Remodeling of the pulmonary artery in idiopathic pleuroparenchymal fibroelastosis
Source: Sci Rep. 2020 Jan 15;10:306. doi: 10.1038/s41598-019-57248-3 (PMC6962210; doi:10.1038/s41598-019-57248-3)
Supplement: Supplementary file 2 — Supplementary data 1. Respiratory function data. [file 41598_2019_57248_MOESM2_ESM.pdf]

SUPPLEMENTARY INFORMATION

Remodeling of the pulmonary artery in idiopathic pleuroparenchymal fibroelastosis

Yoshiaki Kinoshita, Hiroshi Ishii, Hisako Kushima, Takeshi Johkoh, Hidetake Yabuuchi, Masaki Fujita, Kazuki Nabeshima, and Kentaro Watanabe

Supplementary data 1. Respiratory function data

We evaluated the respiratory functional parameters that were measured less than one year prior to the histologic examinations. The forced vital capacity (FVC) and forced expiratory volume in 1 second (FEV_1) were obtained using spirometry. The total lung capacity (TLC) and residual volume (RV) were obtained using the helium gas dilution method, and the diffusing capacity for carbon monoxide (DL_{CO}) was obtained using the single-breath-hold method [1]. Each predicted value was calculated using the formula of the Japanese Respiratory Society [2]. The respiratory functional data were expressed as absolute values (ml) and percentages of predicted values (% pred).

References

1. Forster RE, Fowler WS, Bates D V, et al. The absorption of carbon monoxide by the lungs during breath-holding. *J. Clin. Invest.* 1954; **33**; 1135-1145.
2. Japanease Respiratory Society Guidelines for respiratory function tests, Medical Review Publishers, Tokyo, 2004.
